# CB1R-Mediated Activation of Caspase-3 Causes Epigenetic and Neurobehavioral Abnormalities in Postnatal Ethanol-Exposed Mice

**DOI:** 10.3389/fnmol.2018.00045

**Published:** 2018-02-20

**Authors:** Shivakumar Subbanna, Nagaraja N. Nagre, Madhu Shivakumar, Vikram Joshi, Delphine Psychoyos, Abdullah Kutlar, Nagavedi S. Umapathy, Balapal S. Basavarajappa

**Affiliations:** ^1^Division of Analytical Psychopharmacology, Nathan Kline Institute for Psychiatric Research, New York, NY, United States; ^2^Institute of Biosciences and Technology, Texas A&M University Health Science Center, Houston, TX, United States; ^3^Center for Blood Disorders, Augusta University, Augusta, GA, United States; ^4^New York State Psychiatric Institute, New York, NY, United States; ^5^Department of Psychiatry, College of Physicians & Surgeons, Columbia University, New York, NY, United States; ^6^Department of Psychiatry, New York University Langone Medical Center, New York, NY, United States

**Keywords:** MeCP2, FASD, neurodegeneration, DNA methylation, synaptic plasticity

## Abstract

Alcohol exposure can affect brain development, leading to long-lasting behavioral problems, including cognitive impairment, which together is defined as fetal alcohol spectrum disorder (FASD). However, the fundamental mechanisms through which this occurs are largely unknown. In this study, we report that the exposure of postnatal day 7 (P7) mice to ethanol activates caspase-3 via cannabinoid receptor type-1 (CB1R) in neonatal mice and causes a reduction in methylated DNA binding protein (MeCP2) levels. The developmental expression of MeCP2 in mice is closely correlated with synaptogenesis and neuronal maturation. It was shown that ethanol treatment of P7 mice enhanced *Mecp2* mRNA levels but reduced protein levels. The genetic deletion of CB1R prevented, and administration of a CB1R antagonist before ethanol treatment of P7 mice inhibited caspase-3 activation. Additionally, it reversed the loss of MeCP2 protein, cAMP response element binding protein (CREB) activation, and activity-regulated cytoskeleton-associated protein (Arc) expression. The inhibition of caspase-3 activity prior to ethanol administration prevented ethanol-induced loss of MeCP2, CREB activation, epigenetic regulation of Arc expression, long-term potentiation (LTP), spatial memory deficits and activity-dependent impairment of several signaling molecules, including MeCP2, in adult mice. Collectively, these results reveal that the ethanol-induced CB1R-mediated activation of caspase-3 degrades the MeCP2 protein in the P7 mouse brain and causes long-lasting neurobehavioral deficits in adult mice. This CB1R-mediated instability of MeCP2 during active synaptic maturation may disrupt synaptic circuit maturation and lead to neurobehavioral abnormalities, as observed in this animal model of FASD.

## Introduction

Ethanol abuse during pregnancy is considered to be one of the most common causes of non-genetic mental impairments in the Western world (Mattson et al., 2011). It can cause a range of developmental, cognitive, and behavioral problems, which together is known as fetal alcohol spectrum disorder (FASD). FASD is identified through pervasive neuropsychological manifestations (Mattson and Riley, 1998; Mattson et al., 1998), including the disturbance of hippocampus (HP) and neocortex (NC) functions (Mattson et al., 1996b; Clark et al., 2000; Bookstein et al., 2001), which cause learning and memory abnormalities (Mattson et al., 1996a, 1999; Jacobson and Jacobson, 1999; Clark et al., 2000; Kaemingk and Halverson, 2000; Willford et al., 2004; Rasmussen et al., 2006). Rodents are the most frequently used animal model for FASD research, and a major component of neurodevelopment in this species takes place after birth (Tran et al., 2000; Cronise et al., 2001). In rodents, the brain is especially susceptible to ethanol exposure between postnatal days 4 and 10 (P4–10), which is comparable to the third trimester of human pregnancy (Bayer et al., 1993). Hence, for a third trimester equivalent exposure, ethanol must be administered to neonate pups in these species. A single day of binge-like ethanol treatment at P7 elicits widespread caspase-3 activation (CC3, a marker of neurodegeneration) in numerous regions of the brain, such as the HP and NC (Ikonomidou et al., 2000; Saito et al., 2010; Wilson et al., 2011; Sadrian et al., 2012; Subbanna et al., 2013b); it causes persistent deficits in the olfactory and hippocampal pathway (Wilson et al., 2011; Sadrian et al., 2012) and impairs cognitive function in adults (Abel et al., 1983; Bonthius and West, 1991; Bellinger et al., 1999, 2002; Berman and Hannigan, 2000; Alati et al., 2006; Brown et al., 2007; Brady et al., 2013; Subbanna et al., 2013a, 2015a; Subbanna and Basavarajappa, 2014; Basavarajappa, 2015).

In spite of extensive studies (Ikonomidou et al., 2000; Olney et al., 2002b; Hellemans et al., 2010; Saito et al., 2010; Wilson et al., 2011; Sadrian et al., 2012; Subbanna et al., 2013a,b, 2014; Olney, 2014; Subbanna and Basavarajappa, 2014; Nagre et al., 2015), the molecular mechanisms responsible for the developmental catastrophes induced by ethanol are still poorly defined, making it hard to develop effective treatment strategies for FASD. We have previously shown that third trimester equivalent ethanol exposure in P7 mice activated CB1R (Subbanna et al., 2013a, 2015b), and induced global, as well as gene specific, epigenetic changes (Nagre et al., 2015; Subbanna et al., 2015a) that collectively caused neurobehavioral abnormalities in adult animals that are comparable to the cognitive deficits found in human FASD (Mattson et al., 2010, 2011; Weyrauch et al., 2017). A growing array of new research has portrayed many intriguing ways in which the endocannabinoid system (for review, see Basavarajappa, 2007a; Pava and Woodward, 2012), which includes endogenous ligands (endocannabinoids, ECs), receptors and EC catabolizing enzymes (Piomelli, 2003; Basavarajappa, 2007b), has a vital function in controlling neurobehavioral outcome (Ohno-Shosaku et al., 2001; Wilson and Nicoll, 2001; Bacci et al., 2004) in the developing and adult brain (see Basavarajappa et al., 2009, 2017; Basavarajappa, 2015; Henderson-Redmond et al., 2016).

Methylated DNA makes essential contributions to mammalian development and function (Attwood et al., 2002; Bergman and Cedar, 2013; Basavarajappa and Subbanna, 2016; Subbanna et al., 2016) through its regulation of gene expression (Bird, 2002). DNA methylation silences gene expression partly through recruitment of methyl-CpG binding proteins (MeCP2), which selectively associate with methylated cytosine-phospho-guanine (CpG) dinucleotides. Furthermore, MeCP2 silences transcription by recruiting additional repressive factors that regulate histone acetylation and methylation (Jones et al., 1998; Nan et al., 1998; Nielsen et al., 2001), and operates as a mechanistic bridge between DNA and histone methylation, consequently reinforcing the repressive function of these two distinct epigenetic events (Fuks et al., 2003). Thus, genetic ablation of the MeCP2 gene leads to defects in synaptic development and maturation, and a complete loss of MeCP2 function leads to brain development disorders like Rett Syndrome (RTT), which is a severe autism spectrum disorder (Amir et al., 1999; Chen et al., 2001; Moretti et al., 2006). Several studies have suggested that both DNA methylation and DNA-associated histone modification, which are two of the most commonly studied epigenetic modifications that are important in integrating gene expression in various cells (Reik, 2007; Suzuki and Bird, 2008; Ma et al., 2010), are altered by developmental ethanol exposure. Studies show that ethanol exposure during early brain growth leads to genome-wide/gene-specific alterations in DNA-associated histone protein posttranslational modifications (Kim and Shukla, 2005; Park et al., 2005; Pal-Bhadra et al., 2007; Moonat et al., 2013; Subbanna et al., 2013b) and alterations in DNA methylation (Garro et al., 1991; Haycock and Ramsay, 2009; Liu et al., 2009; Ouko et al., 2009; Downing et al., 2011; Zhou et al., 2011), as well as impaired DNA methylation enzymes (Mukhopadhyay et al., 2013; Nagre et al., 2015) and altered long-lasting phenotypes (Subbanna et al., 2016) that resemble those of fetal alcohol syndrome (Kaminen-Ahola et al., 2010). Together, these critical findings suggest that epigenetic responses to developmental ethanol exposure have the capacity to act as efficient epigenetic modulators and induce impairments in neuronal maturation, leading to long-lasting synaptic dysfunction (Izumi et al., 2005; Noel et al., 2011; Wilson et al., 2011; Subbanna et al., 2013a, 2015a; Subbanna and Basavarajappa, 2014). In our recent study (Nagre et al., 2015), ethanol-induced CB1R-mediated caspase-3 activation was shown to reduce DNA methyltransferase (DNMT1 and DNMT3A) protein levels, leading to the loss of DNA methylation. This present study was undertaken to examine the influence of postnatal ethanol treatment on CB1R-mediated caspase-3-facilitated MeCP2 loss and its impact on neurodegeneration and several neurobehavioral abnormalities.

## Materials and Methods

### Experimental Animals

CB1R knockout (KO), and wild-type (WT) male and female littermates were obtained from a CB1R heterozygous breeding colony (C57BL/6J background; Steiner et al., 1999) at Nathan Kline Institute for Psychiatric Research (NKI). C57BL/6J mice or CB1R KO and WT mice that are on the C57BL/6J background were housed in groups in standard cages at 21–24°C with 40%–60% humidity, a 12-h light/12-h dark cycle, and access to food and water *ad libitum*. Animal care and handling practices were approved by the Animal Use Subcommittee of the NKI (NKI IACUC) and followed National Institutes of Health guidelines. The CB1R KO and WT mice were genotyped using polymerase chain reaction (PCR) analysis of genomic DNA taken from the mouse tails as previously described by our laboratory (Basavarajappa et al., 2003).

### Experimental Ethanol Model

Animal models have been widely used to investigate the relationship among ethanol levels, drinking patterns and brain damage (Maier et al., 1999; Maier and West, 2001; Ethen et al., 2009). Some of those studies have demonstrated a nearly linear inverse relationship between ethanol dose and damage to the developing brain (Maier et al., 1999; Maier and West, 2001; Ethen et al., 2009). Because synaptogenesis takes place after birth in mice (Tran et al., 2000; Cronise et al., 2001), we have administered ethanol to neonate pups to mimic third trimester alcohol exposure in humans. In the present study, we followed an ethanol administration paradigm that does not cause lethality and was shown to cause substantial apoptotic neurodegeneration in P7 mice (Olney et al., 2002a). In each litter, half of P7 (based on the day of birth) male and female pups were treated subcutaneously (s.c.) with saline and ethanol (2.5 g/kg s.c. at 0 h and again at 2 h) as described previously (Wilson et al., 2011; Sadrian et al., 2012; Subbanna et al., 2013a,b). The pups and dams remained together until they were sacrificed, and the pup brains were removed 4–24 h after the first saline/ethanol treatment. To determine blood ethanol levels (BELs), the pups were euthanized through decapitation, and truncal blood was taken at 4 and 9 h following the first ethanol injection. The pup serum ethanol concentrations were then analyzed using an alcohol dehydrogenase-based method (Lundquist, 1959).

### Drug Treatment

In our previous studies, administration of a CB1R antagonist (SR141716A; a gift from RBI, Natick, MA, USA) or use of CB1R KO mice prevented P7 ethanol activated caspase-3 (Subbanna et al., 2013a; Subbanna and Basavarajappa, 2014). In the current study, we used SR141716A and CB1R KO mice to block ethanol-induced caspase-3 activation in P7 mice. SR141716A was dissolved in ethanol (10 μl) followed by Tween 80 (10 μl) and the rest of the volume was adjusted with a sterilized saline. The drug (SR) was administered (1 mg/kg) by s.c. injection at a volume of 5 μl/g body weight 30 min before ethanol treatment. The vehicle (ethanol (10 μl), followed by Tween 80 (10 μl) and saline), was administered as a control. The pups remained with the dams till were sacrificed, and the pup brains were removed 8 h after the first saline/ethanol treatment. Intoxication (sleeping time) as a result of P7 ethanol exposure was not affected by SR administration (Subbanna et al., 2013a,b). P7 mice that were treated with SR exhibited no damage to any of the organs (Subbanna et al., 2013a,b). The broad-spectrum caspase inhibitor, quinoline-Val-Asp (Ome)-CH2-O-phenoxy (Q-VD-OPh; SM Biochemicals, Anaheim, CA, USA; 1 mg/kg; Subbanna et al., 2013b) was dissolved in sterile saline and administered by s.c. injection at a volume of 5 μl/g body weight 30 min prior to ethanol treatment. In some experiments, saline, ethanol, saline + Q-VD-OPh and ethanol + Q-VD-OPh treated mice were weaned and allowed to mature to adulthood. Three-month-old male and female mice were used for behavior analysis. Male mice were used for long-term potentiation (LTP) and activity-dependent signaling experiments.

### Protein Extraction, Electrophoresis and Immunoblotting

Four to twenty-four hours after the first saline or ethanol treatment, P7 mice were sacrificed by decapitation. The dissected HP and NC were flash frozen and stored at −80°C. Tissue homogenates containing freshly added phosphatase inhibitors and 1% protease inhibitor mixture (Roche, Indianapolis, IN, USA) were subjected to cytosolic and nuclear fractionation. Nuclear fraction was further processed using nuclear extraction buffer (Grabowski, 2005) in accordance to the manufacturer’s instructions (Thermo Fisher Scientific, Suwanee, GA, USA) as described before (Basavarajappa et al., 2008, 2014; Basavarajappa and Subbanna, 2014). The samples were stored at −80°C. Developmental brain nuclear fractions were prepared by sacrificing P2 to P90 mice according to their date of birth. The western blots were incubated with anti-mouse activity regulated cytoskeleton-associated protein (Arc; monoclonal, # sc17838, 1:1000; Santa Cruz Biotechnology, CA, USA), anti-rabbit MeCP2 (monoclonal, # 3456, 1:1000), anti-rabbit cleaved caspase-3 (CC3; Asp175; polyclonal, #9661, 1:1000), anti-rabbit CREB (monoclonal, #9197, 1:1000) anti-rabbit phospho-CREB (monoclonal, #9198, 1:1000) and anti-mouse β-actin (monoclonal, #3700, 1:1000; Cell Signaling, Danvers, MA, USA) antibodies for 3 h at room temperature or overnight at 4°C and processed (Basavarajappa et al., 2008). Incubation of western blots with a secondary antibody (goat anti-mouse peroxidase conjugate, #AP 124P, 1:5000, Millipore; goat anti-rabbit, #AP132P, 1:5000, Millipore) alone produced no bands.

### Immunohistochemistry

The free-floating sections were prepared according to our previously described protocols (Subbanna et al., 2013a,b). In double-labeling studies, the free-floating brain sections obtained 8 h after the first saline or ethanol treatment were subjected to dual immunofluorescence labeling as described previously (Subbanna et al., 2013a,b). We used anti-mouse 5mC (#BY-MECY-0100, 1:500, Anaspec Inc., Fremont, CA, USA) or anti-rabbit MeCP2 to label methylated cytosine or MeCP2, respectively, in matured neurons (anti-rabbit NeuN, #24307, 1:200, Cell Signaling, Denvers, MA, USA or anti-mouse NeuN, MAB377, 1:200, EMD Millipore, Billerica, MA, USA). Alexa Fluor 488 or 568 conjugated secondary antibodies (Invitrogen, NY, USA) were used in these studies. Anti-rabbit 5mC or anti-rabbit MeCP2 and anti-mouse NeuN antibodies were omitted to determine the specificity of secondary antibodies. The Mander’s correlation coefficient was used to measure the colocalization of 5-mC or MeCP2 with NeuN-positive neurons (Emi et al., 2013; Subbanna et al., 2016) using LSM-880 with Airyscan and Zen Imaging software. Colocalization was analyzed by selecting CA1 and cortex brain regions.

### DNA Methylation Assay and 5-mC Dot Blot

The pups were decapitated at 8 h (the ideal time for maximum caspase-3 activation) after P7 ethanol administration, and the HP and NC tissues were dissected, flash frozen and stored at −80°C. Qiagen DNA Extraction Kit (Qiagen Sciences, Germantown, MD, USA) was used to isolate genomic DNA from the HP and NC tissues. Epigentek (Farmingdale, NY, USA) MethylFlash DNA Methylation Quantification Kit (Colorimetric) was used to determine the global DNA methylation levels (Nagre et al., 2015; Subbanna et al., 2016). The 5-mC levels were determined using the formula provided in the kit and was normalized to the percentage in the saline control (the graphs represent the DNA methylation levels multiplied by an arbitrary factor to set the saline group to 100). For the dot blot assay, DNA samples (200 ng, 100 ng and 50 ng) were dot-blotted onto a 0.2 μm nitrocellulose membrane in a total volume of 1 μl per sample. The membranes were processed as described before by our laboratory (Basavarajappa et al., 2008; Subbanna et al., 2016) and incubated with a primary rabbit anti-5-methylcytosine antibody (1:500, # BY-MECY-0100, AnaSpec Inc., Fremont, CA, USA). The dot blots were normalized to the percentage in the saline-treated samples.

### Quantitative Real-Time Polymerase Chain Reaction (RT-qPCR)

Four to twenty-four hours after the first saline or ethanol treatment, P7 mice were sacrificed by decapitation, and the dissected HP and NC were flash frozen and stored at −80°C. The HP and NC tissues were used for a total RNA preparation using an RNeasy Mini Kit (Qiagen, Valencia, CA, USA) as described before (Subbanna et al., 2013b). RT-qPCR analysis of *Mecp2* was performed (Integrated thermocycler and fluorescence detector ABI PRISM 7900HT Sequence Detector, Applied Biosystems, Thermo Fisher Scientific, Grand Island, NY, USA) using the predeveloped TaqMan^®^ Gene Expression Assays and primer mixtures Mm00521967_m1 (*Mecp2*) and 4352932 (*Gapdh*; Applied Biosystems, Thermo Fisher Scientific, Grand Island, NY, USA). The cDNA was amplified in Stratagene Mx3000P QPCR systems (Agilent Technologies, Santa Clara, CA, USA) by quantitative RT-PCR using SYBR Green PCR Master Mix (Applied Biosystems, Warrington, UK). The *Arc* primer (forward, 5′-GGTGAGCTGAAGCCACA AAT-3′ and reverse, 5′-GCTGAGCTCTGCTCTTCTTCA-3′) was selected from https://mouseprimerdepot.nci.nih.gov. The gene expression profile for *Mecp2* and *Arc* were normalized to the corresponding *Gapdh* and *Hprt* levels respectively for each sample. Experiments with each set of samples were repeated as three separate runs with triplicate reactions. The data were calculated using SDS2.4 software (Applied Biosystems, Thermo Fisher Scientific, Grand Island, NY, USA). The relative expression level of *Mecp2* or *Arc* (fold level) were analyzed using the 2^−ΔΔCt^ method (Livak and Schmittgen, 2001).

### Spatial Memory (SM) Using the Y-maze

A separate cohort of male and female mice (3 months old; *n* = 8/group) treated with saline, ethanol, saline + Q-VD-OPh or ethanol + Q-VD-OPh at P7 were subjected to an Spatial Memory (SM) task (Dellu et al., 1992), which was performed (Sarnyai et al., 2000) using a symmetrical Y-maze precisely as outlined previously (Basavarajappa et al., 2014; Basavarajappa and Subbanna, 2014). Briefly, the entry to one arm (the novel arm) of Y-maze was blocked using a sheet of opaque paper during the training trials (10 min). After a 24 h intertrial interval, the mice were allowed to explore all three arms (3 min, preference trial). The number of arm entries and the time spent in each arm were noted manually from video recordings by an observer blind to the treatment of the mice. The discrimination ratio for arm entries and dwell time was calculated using the following formula [preference for the novel arm over the familiar other arm (Novel/Novel + Other)].

### Spontaneous Alternation on Y Maze

Individual mouse was placed in the center of the Y maze and was allowed to explore freely through the maze during an 8 min session. The sequence, time spent in each arm and total number of arms entered was recorded. Complete arm entry was considered when the hind paws of the mouse had been totally placed in the arm. Percentage alternation is calculated as described before by our laboratory (Basavarajappa et al., 2014; Basavarajappa and Subbanna, 2014).

### Social Recognition Memory (SRM)

A separate cohort of male mice (3 months old; *n* = 8/group) treated with saline, ethanol, saline + Q-VD-OPh or ethanol + Q-VD-OPh at P7 were subjected to an Social Recognition Memory (SRM) task (Thor et al., 1982; Kogan et al., 2000) that was performed exactly as described earlier (Subbanna and Basavarajappa, 2014). The experimental cages used for this task were the same as those in which the mice were housed (plastic, 27 cm long × 16 cm wide × 12 cm high). In brief, animals were habituated to the new experimental cage by placing an individual adult mouse (male or female) in the cage for 15 min. Immediately after the 15 min of habituation, a juvenile mouse (same sex as the adult; 3–4 weeks old) was introduced into the experimental cage with the adult for an initial contact trial of 2 min. The animals were placed back in their original home cages during the intertrial interval (24 h periods). After 24 h (intertrial delay), the aforementioned juvenile mouse was placed back into the adult’s cage in order to conduct a 2-min test trial. The duration of the social investigation (with a hand-held stopwatch) was recorded by a trained observer. The social investigative behaviors, such as direct interaction with the juvenile while checking any part of the body (including grooming, licking and pawing); sniffing of the mouth, ears, tail, anogenital region; and close following (within 1 cm) of the juvenile (Thor et al., 1982; Kogan et al., 2000), were recorded. The adult mouse that failed to investigate the young mouse for a minimum of 24 s during the initial trial (i.e., 20% of the test time) was re-examined once with another juvenile. The sessions with initial investigation times less than 24 s or any aggressive encounter between the animals were eliminated and the social investigation (%) was calculated as described previously (Basavarajappa et al., 2014; Basavarajappa and Subbanna, 2014; Subbanna and Basavarajappa, 2014; Subbanna et al., 2016).

### LTP

Male mice (3 months old; *n* = 5/group) treated with saline, ethanol, saline + Q-VD-OPh or ethanol + Q-VD-OPh at P7 were sacrificed by cervical dislocation followed by decapitation. HP slices (400 μm) were prepared as described before (Subbanna et al., 2013a). Electrophysiological recording was performed using a standard procedure as described earlier (Subbanna et al., 2013a; Basavarajappa et al., 2014; Basavarajappa and Subbanna, 2014; Subbanna and Basavarajappa, 2014). Briefly, following cutting, slices were placed in a recording chamber (29°C) and perfused with artificial cerebrospinal fluid (ACSF in mM: 124.0 NaCl, 4.4 KCl, 1.0 Na_2_HPO_4_, 25.0 NaHCO_3_, 2.0 MgSO_4_, 2.0 CaCl_2_, 10.0 glucose, osmolarity 290–300) continuously bubbled with 95% O_2_ and 5% CO_2_. The stimulating and the recording electrodes were placed in the CA1 *stratum radiatum* and CA1 field-excitatory-post-synaptic potentials (fEPSPs) were recorded. The stimulus voltages were plotted against the fEPSP slopes to determine basal synaptic transmission (BST). A baseline was recorded up to 10 min at an intensity that evokes approximately 35% of the maximum evoked response. LTP (four pulses at 100 Hz, with the bursts repeated at 5 Hz, and each tetanus including 3 × 10-burst trains separated by 15 s) was induced by tetanic stimulation of the Schaeffer collateral pathway. Responses were recorded for 2 h after, and measured as the fEPSP slope expressed as a percentage of the baseline.

### Activity-Dependent Regulation of Signaling Events and Arc Expression

A separate cohort of male mice (3 months old; *n* = 8/group) treated with saline, ethanol, saline + Q-VD-OPh or ethanol + Q-VD-OPh at P7 were subjected to an SM task (Dellu et al., 1992), which was performed (Sarnyai et al., 2000) using the symmetrical Y-maze (SM) exactly as outlined before (Basavarajappa et al., 2014; Basavarajappa and Subbanna, 2014). After a 24 h intertrial interval, when the mice completed exploring all three arms (3 min, preference trial; test trial), the mice were sacrificed immediately. Hippocampi were collected and processed for analysis of several signaling molecule levels using western blotting, as described previously. The western blots were incubated with anti-rabbit MeCP2 (monoclonal, # 3456, 1:1000, Cell Signaling Technology, Danvers, MA, USA), anti-mouse phospho-MeCP2 (# PIPA535396, 1:1000, Fischer Scientific, Boston, MA, USA, anti-mouse CamKIV (monoclonal, # 610275, 1:1000, BD Biosciences, San Jose, CA, USA), anti-rabbit phospho-CaMKIV (Polyclonal # sc28443, 1:1000), anti-mouse Arc (monoclonal, # sc17838, 1:1000; Santa Cruz Biotechnology, CA, USA), anti-rabbit CREB (monoclonal, #9197, 1:1000), anti-rabbit phospho-CREB (monoclonal, #9198, 1:1000), and anti-mouse β-actin (monoclonal, #3700, 1:1000; Cell Signaling Technology, Denvers, MA, USA) antibodies for 3 h at room temperature, or overnight at 4°C, and processed as previously described by our laboratory (Basavarajappa et al., 2008). Western blots incubated with a secondary antibody (goat anti-mouse peroxidase conjugate, #AP 124P, 1:5000, Millipore; goat anti-rabbit, #AP132P, 1:5000, Millipore) alone produced no bands.

### Chromatin Immunoprecipitation (ChIP) Assay

ChIP was performed as described previously (Subbanna et al., 2014, 2015a). Briefly, adult mouse HP tissue (25 mg) was fixed with 1% formaldehyde, homogenized and subjected to DNA shearing; the amount of sample was normalized to contain equivalent protein amounts. An aliquot of precleared chromatin was used as the input. Chromatin was immunoprecipitated with antibody recognizing normal rabbit IgG as a negative control and with anti-H3K9me2 and anti-H3K14ace antibodies. The quantification of immunoprecipitated DNA and normalization vs. total DNA (input) were performed using real-time qPCR with mouse primers (forward, 5′-AGTGCTCTGGCGAGTAG TCC-3′ and reverse, 5′-TCGGGACAGGCTAAGAACTC-3′) designed to amplify short regions of the Arc gene promoter. Relative quantification of H3K9me2- and H3K14ace-associated genes in saline and treatment groups was performed using the ΔΔCt method (Schmittgen and Livak, 2008).

### Statistical Analysis

The experiments were performed and assessed with an equal number of P7 or P90 mice/treatment. One-way analysis of variance (ANOVA) or two-way ANOVA followed by Bonferroni’s *post hoc* test was used for a statistical comparison of the data and performed using Prism software (GraphPad, San Diego, CA, USA). The level of significance was set at *p* < 0.05, and all results are reported as the mean ± SEM of at least three independent experiments.

## Results

### P7 Ethanol Treatment Activates Caspase-3 and Reduces DNA Methylation and MeCP2 Protein Levels in the Neonatal Brain

Ethanol (2.5 g/kg, s.c. at 0 h and again at 2 h) treatment of P7 mice resulted in BELs of 0.38 ± 0.3 g/dl at 4 h that steadily reached 0.22 ± 0.05 g/dl at 9 h after the first ethanol administration. We performed IHC using CC3 antibody and found CC3 staining throughout the forebrain. Western blot analysis was also performed in saline, 8 h and 24 h ethanol groups and the results suggested that a significantly higher CC3 protein levels in both the HP and NC tissues (data not shown) as observed in our previous studies (Subbanna et al., 2013a,b; Nagre et al., 2015). Thus, binge-like ethanol treatment of P7 mice recapitulated neurodegeneration conditions as observed previously (Ikonomidou et al., 2000; Subbanna et al., 2013a).

**Figure 1:**
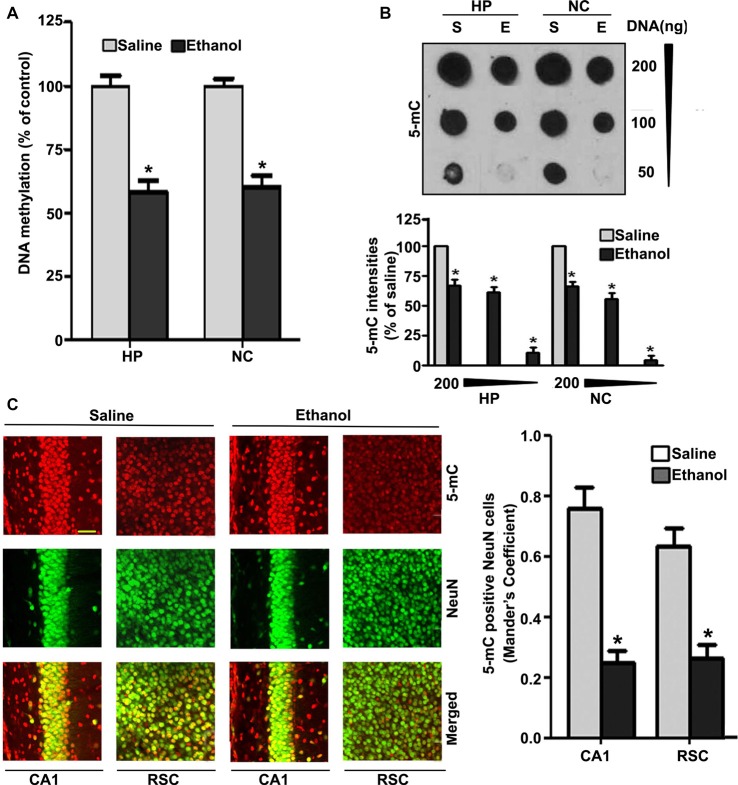
Treatment of postnatal day 7 (P7) mice with ethanol reduces DNA methylation in the brain. **(A)** Global DNA methylation was reduced by ethanol treatment. Global DNA methylation quantification in DNA from the hippocampus (HP) and neocortex (NC) obtained 8 h after the first saline or ethanol injection. For the 0 h ethanol group, saline was injected. (*p < 0.05, *n* = 10 pups/group). **(B)** Global 5-mC-specific dot-blot intensities of genomic DNA prepared from the HP and NC (200 ng, 100 ng and 50 ng of genomic DNA) of P7 mice after treatment with saline or ethanol (8 h). The 5-mC-specific dot-blot intensities (%) compared with the saline controls (*p < 0.01 vs. Saline, *n* = 8 pups/group). **(C)** Free-floating coronal brain sections (HP and RSC) from both the groups (saline and 8 h ethanol) were subjected to immunohistochemistry with anti-mouse 5mC and anti-rabbit NeuN antibodies to label 5-mC-positive NeuN in neurons. Mander’s coefficient analysis was used to evaluate 5-mC-positive NeuN neurons in the CA1 and RSC brain regions. (*p < 0.01 vs. Saline, *n* = 6 pups/group). Scale bars = 10 μm. Error bars, SEM (one-way analysis of variance (ANOVA) with Bonferroni’s *post hoc* test).

Because degenerating neurons in ethanol-exposed P7 mice undergo nuclear changes that lead to caspase-3-mediated loss of DNA methyltransferases (DNMT1 and DNMT3A), and thus reduced DNA methylation (Nagre et al., 2015), we examined DNA methylation status and MeCP2 levels in ethanol-treated P7 mice. Consistent with our previous observation (Nagre et al., 2015), we found that P7 ethanol treatment significantly reduced DNA methylation (HP, *F*_(1,36)_ = 92, *p* < 0.01; NC, *F*_(1,36)_ = 80, *p* < 0.01; one-way ANOVA; Figure 1A). We found a significant drop in the amount of 5-mC in genomic DNA from the HP (*F*_(1,46)_ = 55, *p* < 0.01) and NC (*F*_(1,46)_ = 60, *p* < 0.01; one-way ANOVA with Bonferroni’s *post hoc* tests) of ethanol-treated P7 mice compared with the saline-treated mice (Figure 1B), as determined by dot blot. The dot blots were normalized to the percentage of 5-mC in the saline-treated mouse samples (the graphs represent the 5-mC levels multiplied by an arbitrary factor to set the saline-treated sample levels to 100). IHC of 5-mC and NeuN, followed by Mander’s coefficient analysis, suggests that ethanol treatment significantly reduced the number of 5-mC-positive NeuN neurons compared with saline treatment in both CA1 (*F*_(3,28)_ = 29, *p* < 0.01) and retrosplenial cortex (RSC; *F*_(3,28)_ = 32, *p* < 0.01; one-way ANOVA with Bonferroni’s *post hoc* tests) regions (Figure 1C).

**Figure 2:**
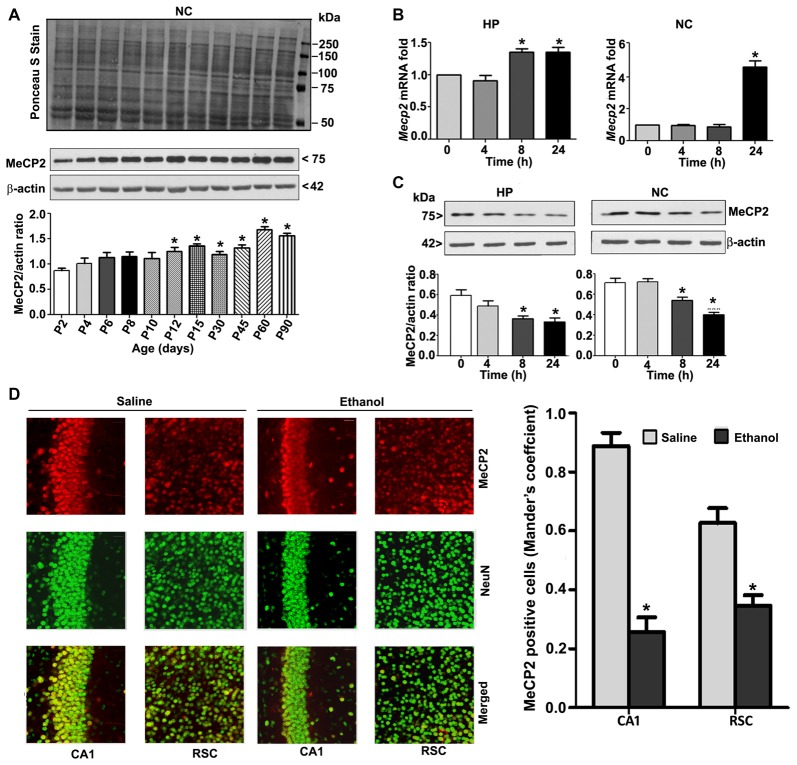
MeCP2 protein expression pattern during mouse brain development; ethanol treatment of P7 mice enhances Mecp2 mRNA but impairs the protein levels in the HP and NC regions. **(A)** MeCP2 protein expression pattern during mouse brain development. The MeCP2 expression pattern in neocortical nuclear protein extract obtained from P2 to P90 mouse brains were subjected to western blotting. Equal protein loading was confirmed after Ponceau S staining, and β-actin was used as the protein loading control. (*p < 0.01 vs P2 groups; *n* = 12 pups/group). **(B)** RT-qPCR analysis of *Mecp2* mRNA in HP and NC extracts obtained 4–24 h after the first saline or ethanol injection (*n* = 12 pups/group). *Gapdh* mRNA was used as the internal control for normalization of *Mecp2* mRNA levels. **(C)** Western blot analysis of MeCP2 proteins in the P7 HP and NC nuclear protein extracts (4–24 h after the first saline or ethanol treatment). β-actin was used as the protein loading control. In the 0 h ethanol group, saline was injected [*p < 0.01 vs. saline (0 h) group]. (*n* = 12 pups/group). **(D)** The coronal brain sections (HP, hippocampus, and RSC, retrosplenial cortex; saline and 8 h ethanol) were used for immunohistochemistry with anti-mouse MeCP2 and anti-rabbit NeuN antibodies to label MeCP2-positive NeuN in neurons. Mander’s coefficient analysis was used to evaluate MeCP2-positive NeuN neurons in the CA1 and RSC brain regions. Error bars, SEM (*p < 0.01 vs. Saline, *n* = 6 pups/group). Scale bars = 10 μm. Error bars, SEM (one-way ANOVA with Bonferroni’s *post hoc* test).

To understand the developmental pattern of MeCP2 expression in the mouse brain, we determined the MeCP2 protein levels at various developmental stages. NC nuclear protein extracts from P2 to P90 developmental stages were subjected to western blotting. MeCP2 protein levels were significantly (*F*_(8,45)_ = 80, *p* < 0.05) elevated through synaptogenesis and up to the adulthood (P90; Figure 2A) levels compared with the P2 developmental stage. Next, we tested the effects of ethanol treatment of P7 mice on MeCP2 expression in the HP and NC. We determined the mRNA levels, and the results revealed that *Mecp2* mRNA levels increased at the 8 h and 24 h time points in the HP (*F*_(3,28)_ = 13, *p* < 0.01) and were enhanced at 24 h in the NC (*F*_(3,28)_ = 80, *p* < 0.01; Figure 2B) after administration of the first ethanol dose. The levels of the endogenous mRNA control *Gapdh* were not significantly altered at any time point (data not shown), and *Gapdh* mRNA values were used to normalize *Mecp2* gene expression data. In our next experiment, we used western blotting to measure protein levels. Ethanol treatment decreased MeCP2 protein levels in the HP (*F*_(3,28)_ = 7, *p* < 0.01; 4–24 h) and NC (*F*_(3,28)_ = 19, *p* < 0.01) at the 8–24 h (after the first ethanol injection) time points when compared with the saline control (0 h; Figure 2C). IHC of MeCP2 and NeuN followed by Mander’s coefficient analysis indicated that ethanol treatment significantly (*p* < 0.01) reduced the number of MeCP2-positive NeuN neurons compared with saline treatment in both CA1 (*F*_(3,28)_ = 23, *p* < 0.01) and RSC (*F*_(3,28)_ = 27, *p* < 0.01) regions (one-way ANOVA with Bonferroni’s *post hoc* tests; Figure 2D). These results suggest that the net loss of MeCP2 proteins seen in ethanol-exposed mice may be due to proteolytic degradation.

### Pharmacological Blockade or Genetic Deletion of CB1R Prevents the Ethanol-Induced Reduction in MeCP2 Protein Levels

**Figure 3:**
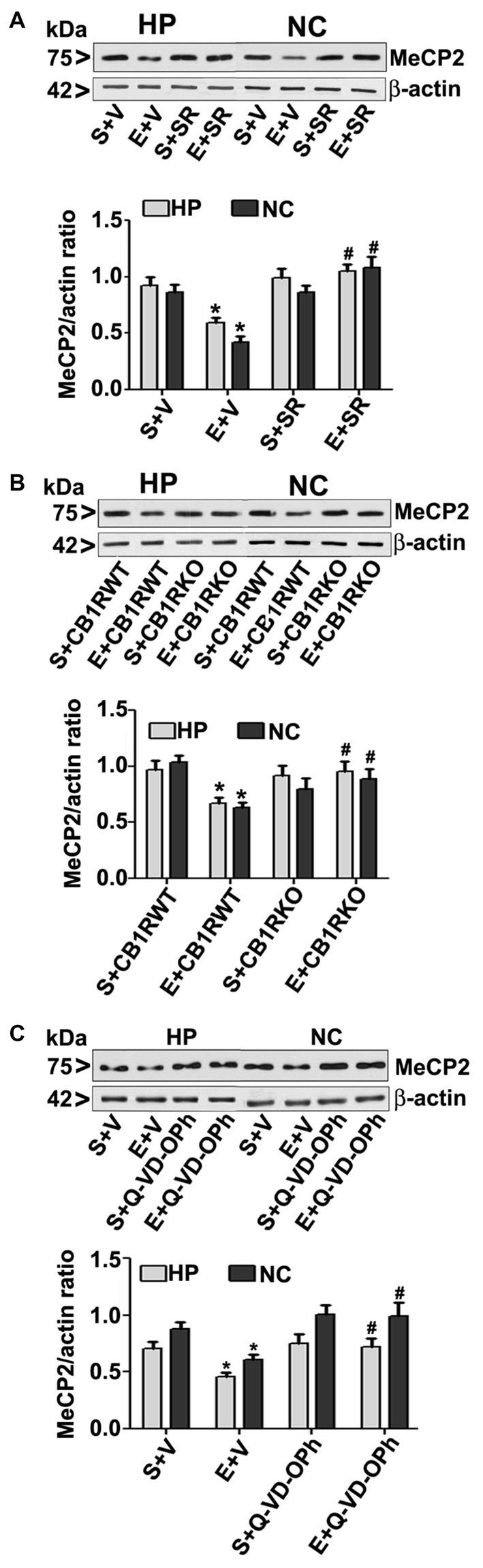
CB1R antagonist (SR141716A) or CB1R knockout (KO) mice or Q-VD-OPh protect against ethanol-mediated loss of MeCP2 proteins. **(A)** P7 mice were pretreated with SR (1 mg/kg) 30 min before saline or ethanol treatment. MeCP2 proteins from hippocampal (HP) and neocortical (NC) nuclear extracts from the four treatment groups (S + V, E + V, S + SR and E + SR) groups (*n* = 12 pups/group) were subjected to western blotting. S, saline; E, ethanol. β-actin was used as the protein loading control. (*p < 0.01 vs. S + V; ^#^*p* < 0.01 vs. E + V). **(B)** P7 CB1R wild-type (WT) and CB1R KO mice were treated with saline or ethanol. From nuclear extracts of HP and NC, western blot analysis of MeCP2 protein was performed for all the four treatment groups (S + CB1R WT, E + CB1R WT, S + CB1R KO + and E + CB1R KO) groups (*n* = 12 pups/group). S, saline; E, ethanol. (*p < 0.01 vs. S + CB1RWT; ^#^*p* < 0.01 vs. E + CB1RWT). **(C)** P7 mice pre-treated for 30 min with Q-VD-OPh (1 mg/kg) or vehicle were exposed to ethanol for 8 h, and MeCP2 protein levels were examined in nuclear extracts of the HP and NC from saline (S), ethanol (E), S + Q-VD-OPh, or E + Q-VD-OPh groups (*n* = 12 pups/group) via western blot analysis. β-actin was used as the protein loading control (*p < 0.01 vs. S; ^#^*p* < 0.01 vs. E. Error bars, SEM (two-way ANOVA with Bonferroni’s *post hoc* test).

To further evaluate whether the loss of MeCP2 proteins is due to ethanol-induced caspase-3 activation, we used a specific CB1R antagonist (SR141716A, SR), CB1R KO mice. In our previous studies (Subbanna et al., 2013a, 2015a), both SR and CB1R KO hinders ethanol-induced activation of caspase-3 without significantly influencing ethanol metabolism. In our previous studies (Subbanna et al., 2013a), SR dose-dependently inhibited the ethanol-induced activation of caspase-3 (CC3 levels), with maximum inhibition at 1 mg/kg (Subbanna et al., 2013a, 2015a). Thus, we used SR at 1 mg/kg in our current studies. Our results suggested that SR pre-administration completely rescued the ethanol-induced decrease in MeCP2 protein levels in the HP and NC (*p* > 0.01; Figure 3A) of P7 mice. Two-way ANOVA with Bonferroni’s *post hoc* analysis suggested that there were significant effects of ethanol (vs. saline; HP: *F*_(1,20)_ = 23, *p* < 0.01; NC: *F*_(1,20)_ = 18, *p* < 0.01) and a significant interaction between ethanol and SR (HP: *F*_(1,20)_ = 13, *p* < 0.01; NC: *F*_(1,20)_ = 18, *p* < 0.01). Treatment with SR or a vehicle alone had no significant effects on MeCP2 protein levels in the absence of, or following ethanol treatment (*p* > 0.05). One-way ANOVA with Bonferroni’s *post hoc* analysis for MeCP2 protein levels in saline- and ethanol-treated P7 CB1R KO and WT mice demonstrated that, consistent with the SR pretreatment results, CB1R KO provided protection against the P7 ethanol-induced decrease in MeCP2 protein levels in the HP (*F*_(1,20)_ = 16, *p* < 0.01) and NC (*F*_(1,20)_ = 20, *p* < 0.01; Figure 3B). Together, these results suggest that a blockade or genetic ablation of CB1Rs prevents the ethanol-induced loss of MeCP2 protein in the HP and NC of the P7 mouse brain through caspase-3 activation.

### Inhibition of Caspase-3 Activity before Ethanol Treatment Prevents the Ethanol-Induced Reduction in MeCP2 Protein Levels

The loss of MeCP2 protein provoked us to further examine the possibility of caspase-3-mediated degradation; therefore, we used Q-VD-OPh in our experiments. Q-VD-OPh prevents caspase-3 cleavage into its 17 kDa active fragment *in vivo* in P7 rats (Renolleau et al., 2007) and mice (Subbanna et al., 2013b). This compound is significantly more effective and less toxic than the widely used caspase-3 inhibitors in preventing apoptosis (for references see; Renolleau et al., 2007; Subbanna et al., 2013b). In our previous studies (Subbanna et al., 2013b; Nagre et al., 2015), we found that Q-VD-OPh rescues the ethanol-induced formation of CC3 and prevents dimethylated histone H3 lysine 9 (H3K9me2), total histone H3, DNMT1 and DNMT3A protein degradation in the HP and NC. Q-VD-OPh (1 mg/kg, s.c.) administration prior to ethanol exposure of P7 mice did not affect the BELs (0.36 ± 0.2 g/dl at 4 h, steadily reduced to 0.24 ± 0.06 g/dl at 9 h after the first ethanol exposure), suggesting that Q-VD-OPh does not significantly change ethanol metabolism. We found that Q-VD-OPh rescued the ethanol-induced loss of MeCP2 protein in the HP (*F*_(1,20)_ = 18, *p* < 0.01) and NC (*F*_(1,20)_ = 19, *p* < 0.01; Figure 3C). The Q-VD-OPh treatment had no significant effect in the saline group (*p* > 0.05). Taken together, these results suggest that the ethanol-induced loss of MeCP2 occurs because of MeCP2 protein degradation induced by activated caspase-3.

### Pre-Treatment of P7 Mice with Q-VD-OPh Rescues the Impaired pCREB and Arc Protein Expression Observed in Ethanol-Exposed P7 and Adult Mice

**Figure 4:**
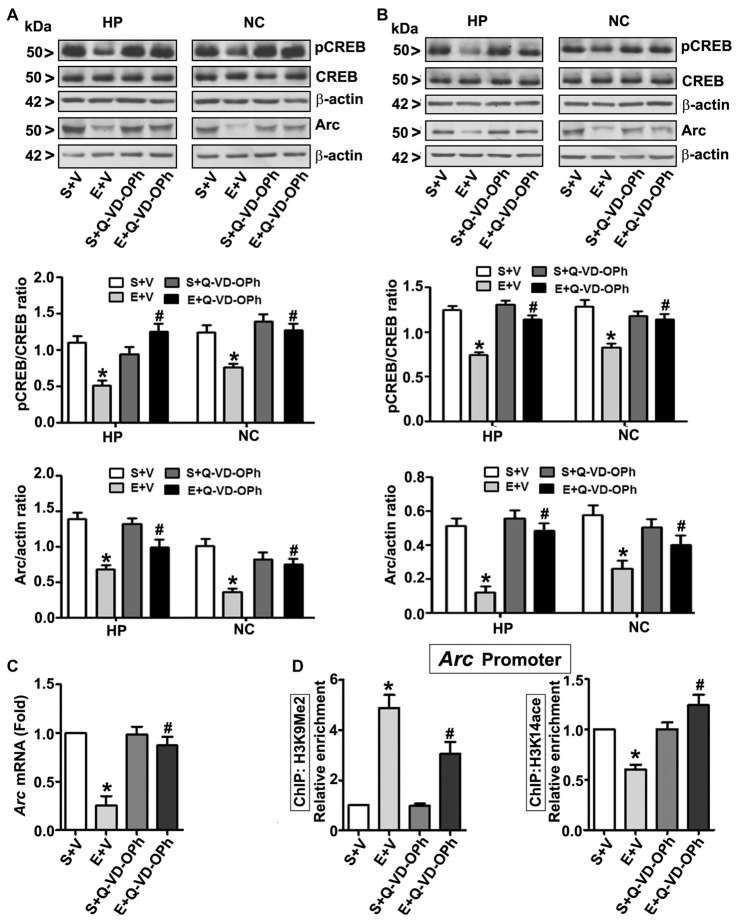
Pharmacological blockades of caspase-3 activation protects against ethanol-induced inhibition of CREB phosphorylation and epigenetic-mediated activity regulatedcytoskeleton-associated protein (Arc) expression in the mouse brain. P7 pups were injected with Q-VD-OPh (1 mg/kg) 30 min before first saline or ethanol exposure and were either sacrificed after 8 h or were allowed to mature to adulthood and then sacrificed. **(A)** P7 and **(B)** adult hippocampal (HP) and neocortical (NC) nuclear extracts from the four treatment groups (S + V, E + V, S + Q-VD-OPh, or E + Q-VD-OPh) were subjected to western blotting to analyze the levels of pCREB, CREB and Arc (*n* = 10 pups/group). The representative blots are shown for the hippocampal and cortical nuclear extracts (*p < 0.01 vs. S + V; ^#^*p* < 0.01 vs. E + V). β-actin was used as the loading control. **(C)** Adult hippocampal nuclear extracts from the four treatment groups (S + V, E + V, S + Q-VD-OPh, or E + Q-VD-OPh) were subjected to RT-qPCR to analyze the *Arc* mRNA levels. *Hprt* mRNA was used as the internal control for normalization of *Arc* mRNA. **(D)** Epigenetic analysis at the promoter region of the *Arc* gene. ChIP analysis of the *Arc* gene promoter in HP tissues from the four treatment groups (S + V, E + V, S + Q-VD-OPh, or E + Q-VD-OPh) with anti-acetylated H3K14 or anti-H3K9me2 antibodies. Levels of *Arc* gene promoter chromatin enrichment in the IPs were measured by RT-qPCR. Error bars, SEM (two-way ANOVA with Bonferroni’s *post hoc* test).

In order to examine the involvement of intracellular signaling events in the long-lasting effects of ethanol on neurodevelopment even further, we ascertained the levels of pCREB (activated), CREB (total) and Arc proteins at 8 h post ethanol treatment. Two-way ANOVA with Bonferroni’s *post hoc* analysis suggested significant effects of ethanol (vs. saline; HP: *F*_(3,33)_ = 43, *p* < 0.01; NC: *F*_(3,33)_ = 38, *p* < 0.01) and a significant interaction between ethanol and Q-VD-OPh (HP: pCREB, *F*_(3,33)_ = 23, *p* < 0.01; Arc, *F*_(3,33)_ = 33, *p* < 0.01 and NC: pCREB, *F*_(3,33)_ = 28, *p* < 0.01; Arc, *F*_(3,33)_ = 38, *p* < 0.01) in P7 mice. Q-VD-OPh or saline alone had no significant effects on pCREB and Arc protein levels (*p* > 0.05) in P7 mice (Figure 4A). To further evaluate whether pCREB and Arc protein expression lasts into adulthood and could be rescued by pre-administration of Q-VD-OPh at P7, we determined pCREB and Arc protein expressions in HP and NC tissues derived from adult mice. Two-way ANOVA with Bonferroni’s *post hoc* analysis indicated that the ethanol effects on pCREB and Arc are long-lasting and are significantly reduced in P7 ethanol-treated adult mice (vs. saline) (HP: *F*_(3,33)_ = 43, *p* < 0.01; NC: *F*_(3,33)_ = 41, *p* < 0.01) and that a significant interaction between ethanol and Q-VD-OPh occurred (HP: pCREB, *F*_(3,33)_ = 20, *p* < 0.01; Arc, *F*_(3,33)_ = 33, *p* < 0.01 and NC: pCREB, *F*_(3,33)_ = 23, *p* < 0.01; Arc, *F*_(3,33)_ = 28, *p* < 0.01). P7 Q-VD-OPh or saline treatment had no significant effects on pCREB and Arc protein levels (*p* > 0.05) in adult mice (Figure 4B).

### Ethanol-Induced Reduction in Arc Expression in Adult Mice Is Regulated by an Epigenetic Mechanism and Rescued by Pre-administration of Q-VD-OPh in P7 Mice

Next, we determined *Arc* mRNA levels in the adult HP. Consistent with the protein expression results (Figure 4B), ethanol exposure at P7 significantly (*F*_(3,33)_ = 44, *p* < 0.01; two-way ANOVA with Bonferroni’s *post hoc*) reduced *Arc* mRNA levels in the adult HP (Figure 4C), and pre-administration of Q-VD-OPh at P7 rescued it, suggesting that long-lasting Arc expression deficits are transcriptionally regulated. It is well established that MeCP2 silences gene transcription by recruiting the histone deacetylase (HDAC, which removes acetyl groups from histones; Nan et al., 1997, 1998) or G9a (adds methyl groups to histones; Fuks et al., 2003; Coward et al., 2017) repressive molecular machineries. Additionally, reduced pCREB levels can impair recruitment of the CREB binding protein, CBP (intrinsic histone acetyl transferase activity, HAT adds acetyl groups to histones) to gene promoters (Lonze and Ginty, 2002; Lubin et al., 2008). Together these events can modulate H3K9me2 and H3K14ace, typical epigenetic marks linked to repressed and active chromatin (Basavarajappa and Subbanna, 2016), respectively, and alter *Arc* expression. Thus, we analyzed these marks at the *Arc* promoter by ChIP assay. Two-way ANOVA with Bonferroni’s *post hoc* analysis suggested that P7 ethanol treatment significantly increased H3K9me2 levels and decreased H3K14ace at the *Arc* promoter region in the adult HP (vs. saline; *F*_(3,33)_ = 56, *p* < 0.01), and that there was a significant interaction between ethanol and Q-VD-OPh (*F*_(3,33)_ = 22, *p* < 0.01). P7 Q-VD-OPh, or saline treatment, had no significant effects on H3K9me2 levels or H3K14ace at the *Arc* promoter region (*p* > 0.05) in adult mice (Figure 4D).

### Pre-administration of Q-VD-OPh before Ethanol Exposure Rescues Ethanol-Induced Memory Loss in Adult Mice

**Figure 5:**
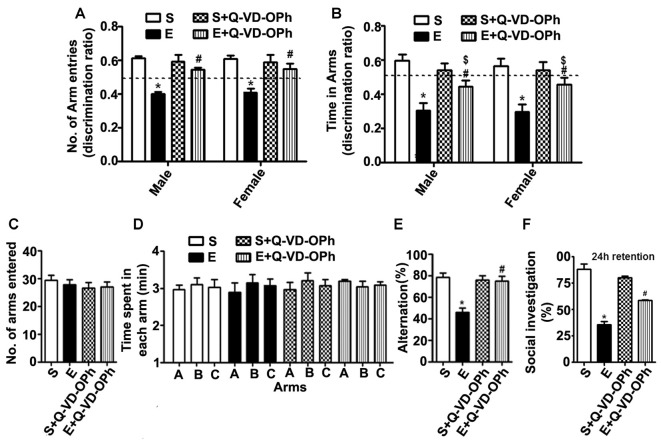
Administration of a caspase-3 inhibitor before ethanol treatment in P7 mice rescues persistent spatial and social recognition memory (SRM) deficits in adulthood. **(A,B)** The spatial working memory was determined using the Y-maze in adult mice treated with saline (S), ethanol (E), S + Q-VD-OPh, or E + Q-VD-OPh at P7. The discrimination ratio [preference for the novel arm over the familiar other arm (Novel/Novel + Other)] for arms entries **(A)** and dwell time (time spent in each arm) **(B)** of S, E, S + Q-VD-OPh and E + Q-VD-OPh -treated mice, 24 h after the first encounter with the partially opened maze. The dashed line denotes chance performance (0.5). **(C–E)** The spontaneous alternation memory of adult mice treated with saline (S), ethanol (E), S + Q-VD-OPh, or E + Q-VD-OPh at P7 was evaluated using the Y-maze. **(C)** The number of arm entries by mice in all the treatment groups. **(D)** The time spent in each arm by mice in all the treatment groups. **(E)** The spontaneous alternation performance by mice in all the treatment groups. **(F)** The percent of social investigation is shown for P7 S, E, S + Q-VD-OPh and E + Q-VD-OPh -treated adult mice, 24 h after the first encounter with the same juvenile mice. (*p < 0.01 vs. saline; ^$^*p* < 0.05 vs. saline; ^#^*p* < 0.05 vs. ethanol, *n* = 8 mice/group). Error bars, SEM (two-way ANOVA with Bonferroni’s *post hoc* test).

To examine whether inhibition of caspase-3 activation in P7 mice prevents the persistent ethanol-induced behavioral deficits observed in our previous behavioral analysis (Subbanna et al., 2013a; Nagre et al., 2015), we tested spatial recognition memory using a Y-maze. Two-way ANOVA analysis suggested that saline- or S + Q-VD-OPh-treated male (arm entry: *F*_(3,33)_ = 29, *p* < 0.01) and female (arm entry: *F*_(3,33)_ = 32, *p* < 0.01) mice entered (Figure 5A) arms more frequently and spent more time (Figure 5B) in (dwell time: male, *F*_(3,33)_ = 8, *p* < 0.01, and female, *F*_(3,33)_ = 10, *p* < 0.01) the novel, previously unvisited arm of the maze. In contrast, P7 ethanol-treated male and female mice exhibited a lower preference toward the novel arm (*p* < 0.01) and spent less time (dwell time; *p* < 0.01) in the novel arm compared with the saline-treated mice after 24 h of retention. Pre-administration of Q-VD-OPh significantly rescued the ethanol-induced impairments (E + Q-VD-OPh), with a preference toward exploration of the novel arm (*p* < 0.01) and increased time spent (*p* < 0.01) in the novel arm. These findings suggest that inhibition of caspase-3 before P7 ethanol treatment ameliorates the spatial recognition memory defect in adult male and female mice. Due to the lack of gender effects, we used only male mice in our remaining adult studies.

In our second behavioral test to examine whether Q-VD-OPh rescues ethanol-induced memory impairments, we tested a spontaneous alternation in the Y maze (Lalonde, 2002) in male adult mice. P7 S, E, S + Q-VD-OPh and E + Q-VD-OPh treatment had no significant effect on the adult exploratory activities, as assessed by the number of arm entries (Figure 5C) and time spent (Figure 5D) in each arm during the Y-maze test. Two-way ANOVA with Bonferroni’s *post hoc* analysis suggested that the ethanol-treatment (E group) exhibited significantly diminished spontaneous alternation compared with saline-treatment (S group) as found in our previous observations (Subbanna et al., 2013a). Pre-administration of Q-VD-OPh before ethanol (E + Q-VD-OPh group) rescued these deficits (*F*_(3,33)_ = 23, *p* < 0.01; Figure 5E). Pre-administration of Q-VD-OPh before saline treatment (S + Q-VD-OPh group) failed to significantly affect the spontaneous alternation performance (*p* > 0.05). Altogether, these results indicated that inhibition of caspase-3 activity prevents the impact of ethanol exposure at P7 on spatial memory defects in adult mice.

### P7 Pre-administration of Q-VD-OPh before Ethanol Treatment Rescues Ethanol-Induced Social Recognition Memory Loss in Adult Mice

Consistent with our previous observations (Subbanna and Basavarajappa, 2014; Subbanna et al., 2015a), the social investigation performance of P7 ethanol-treated adult mice was significantly reduced compared with that of the saline-treated mice. Two-way ANOVA analysis suggested that pre-administration of Q-VD-OPh significantly rescued the ethanol-induced (E + Q-VD-OPh; *F*_(3,33)_ = 10, *p* < 0.01) SRM deficits compared with those of the saline or S + Q-VD-OPh treated mice (Figure 5F). Importantly, S + Q-VD-OPh alone had no significant effect (*p* > 0.05) on SRM, and these mice exhibited normal social interactions with juvenile mice when compared with the saline-treated mice (*p* > 0.05). These results indicate that caspase-3 inhibition before P7 ethanol exposure is effective in rescuing SRM loss in adult mice.

### Q-VD-OPh Pre-administration before Ethanol Treatment Prevents the Ethanol-Induced LTP Deficits

**Figure 6:**
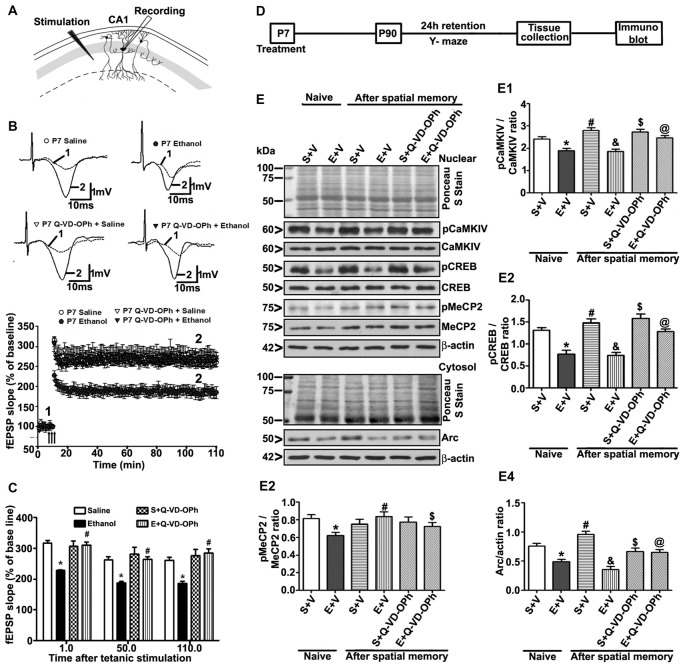
Administration of a caspase-3 inhibitor before P7 ethanol treatment prevents long-term potentiation (LTP) deficits in adult male mice. **(A)** A schematic drawing shows the stimulating and recording electrode positions in the CA1 region of the HP. **(B)** The average field-excitatory-post-synaptic potential (fEPSP) slope at various time points obtained from P7 saline (S), ethanol (E), S + Q-VD-OPh, or E + Q-VD-OPh-treated adult mice. For each slice, the fEPSP slopes were normalized against the average slope over the 10-min recording before LTP stimulation. Arrows show the time of Theta-Burst Stimulation (TBS; four pulses at 100 Hz, with bursts repeated at 5 Hz, and each tetanus including three different 10-burst trains separated by 15 s). **Inset**: representative traces before and after TBS are shown for all the treatment groups. Scale 1 mV; 10 ms. **(C)** Bar graph compares the average of the fEPSP slopes at several time points after TBS in P7 saline (S), ethanol (E), S + Q-VD-OPh, or E + Q-VD-OPh-treated adult mice hippocampal slices. (*p < 0.01 vs. saline****, *n* = 5 mice/group; 10 slices/group). **(D)** Scheme indicating the experimental design to evaluate the effects of pre-administration of Q-VD-OPh before saline/ethanol treatment in P7 mice on activity-dependent signaling events in adulthood. The spatial working memory of adult mice treated with saline (S), ethanol (E), S + Q-VD-OPh, or E + Q-VD-OPh at P7 was evaluated using a Y-maze. **(E)** HP tissues were collected immediately after a 24 h intertrial interval when the mice completed exploring all three arms (3 min, preference trial, test trial). The signaling proteins **(E1)** pCaMKIV, **(E2)** pCREB, **(E3)** pMeCP2 and **(E4)** Arc were examined in nuclear or cytosolic extracts of the HP from saline (S), ethanol (E), S + Q-VD-OPh, or E + Q-VD-OPh groups (*n* = 8 mice/group) via western blot analysis. Equal protein loading was confirmed after Ponceau S staining, and β-actin was used as a protein loading control (*p < 0.01 vs. S; ^#^*p* < 0.01 vs. E; ^&^*p* < 0.01 vs. S+V; ^$^*p* < 0.01 vs S; ^@^*p* < 0.01 vs E. Error bars, SEM (two-way ANOVA with Bonferroni’s *post hoc* test).

To understand whether pre-administration of Q-VD-OPh rescues ethanol-induced LTP deficits, we examined LTP at the Schaffer collateral pathway of hippocampal slices derived from adult male mice treated with saline, ethanol, saline + Q-VD-OPh or ethanol + Q-VD-OPh at P7. First, we measured the input/output (I/O) responses, in which fEPSP slopes were recorded in response to single electrical stimuli of increasing magnitude in adult hippocampal slices. Robust I/O responses (Subbanna et al., 2013a; Subbanna and Basavarajappa, 2014) were observed in all treatment groups and were not altered by ethanol or Q-VD-OPh treatment (*p* > 0.05; data not shown). Prior to Theta-Burst Stimulation (TBS), the baseline fEPSP was recorded at 60 s intervals with a stimulation intensity equivalent to ~35% of the maximum evoked response (10 min). Application of the TBS induced robust LTP in saline-treated animals and was stable over 120 min. However, TBS resulted in substantially weakened LTP in slices derived from P7 ethanol-treated mice (*p* < 0.01). Pre-administration of Q-VD-OPh prior to ethanol treatment rescued the TBS-evoked LTP deficits found in P7 ethanol-treated adult mice (*p* < 0.01) with a significant group interaction (two-way ANOVA; *F*_(1,36)_ = 36, *p* < 0.01; Bonferroni’s *post hoc* analysis; *n* = 10 slices/5 mice/group; Figures 6A,B). Pre-administration of Q-VD-OPh before saline treatment in P7 mice failed to alter LTP responses (*p* > 0.05) in adult mouse hippocampal slices (Figure 6C).

### Treatment of P7 Mice with Q-VD-OPh before Ethanol Exposure Prevents the Ethanol-Induced Deficits in Activity-Dependent Signaling Events and Arc Expression

To examine the function of intracellular signaling events in the long-lasting effects of ethanol even more in-depth, we determined the levels of several signaling molecules (pCaMKIV, pCREB, pMeCP2 and Arc) that are important for activity-dependent development of synaptic circuits (Shieh and Ghosh, 1999; Cohen et al., 2011; Nagendran and Hardy, 2011) in the HP. The schematic in Figure 6D shows the experimental design for this study. Two-way ANOVA with Bonferroni’s *post hoc* analysis suggested significant effects of ethanol (vs. saline; pCaMKIV: *F*_(3,33)_ = 25, *p* < 0.01; pCREB: *F*_(3,33)_ = 22, *p* < 0.01; pMeCP2: *F*_(3,33)_ = 10, *p* < 0.01; Arc: *F*_(3,33)_ = 42, *p* < 0.01) and a significant interaction between ethanol and Q-VD-OPh (pCaMKIV: *F*_(3,33)_ = 18, *p* < 0.01; pCREB: *F*_(3,33)_ = 12, *p* < 0.01; pMeCP2: *F*_(3,33)_ = 9, *p* < 0.01; Arc: *F*_(3,33)_ = 22, *p* < 0.01) in P90 mice. Q-VD-OPh (data not shown) or saline alone had no significant effects on pCaMKIV, pCREB, pMeCP2 and Arc protein levels (*p* > 0.05) in adult naive mice. P7 Q-VD-OPh- (data not shown) or saline-treated mice exhibited enhanced pCaMKIV (Figure 6E1), pCREB (Figure 6E2), pMeCP2 (Figure 6E3) and Arc (Figure 6E4) protein levels (*p* < 0.01) after Y-maze behavior compared with saline-treated naive adult mice. However, P7 ethanol-treated mice failed to enhance pCaMKIV, pCREB, pMeCP2 and Arc protein levels (*p* < 0.01) after Y-maze behavior when compared with P7 ethanol-treated naive adult mice.

## Discussion

In this study, several lines of evidence demonstrate for the first time that postnatal ethanol treatment impairs MeCP2 via CB1R-dependent caspase-3-mediated protein degradation in the P7 mouse brain, causing long-lasting signaling, *Arc* gene expression, and synaptic and behavioral deficits in adult mice. These results support the notion that the MeCP2 protein expression pattern is correlated with neuronal development or maturation and is impaired by postnatal ethanol treatment. A CB1R antagonist rescued and genetic deletion of CB1R prevented loss of MeCP2 protein, and they both are known to prevent the caspase-3 activation (Subbanna et al., 2013a,b, 2015a; Subbanna and Basavarajappa, 2014) in P7 mice. Directly inhibiting caspase-3 with an inhibitor (Q-VD-OPh) also rescued the ethanol-induced loss of MeCP2 protein in P7 mice. In humans and rodents, enhanced MeCP2 levels are present during postnatal development (Balmer et al., 2003; Olson et al., 2014). This developmental expression pattern suggests that MeCP2 can regulate synaptic function (Zoghbi, 2003). MeCP2 is more enriched in the brain than in most other tissues (Lasalle et al., 2001; Shahbazian et al., 2002), is highly expressed in neurons but less in glia (Ballas et al., 2009), and is localized to cell nuclei (Akbarian et al., 2001; Lasalle et al., 2001; Shahbazian et al., 2002). The loss of MeCP2 found in the current study combined with the impaired DNMT1 and DNMT3A expression and DNA methylation in ethanol-treated postnatal mice (Nagre et al., 2015) may lead to long-lasting defects in gene expression, and synaptic and behavioral outcomes in adult animals. Hence, inhibition of caspase-3 activity before ethanol treatment prevented ethanol-induced persistent impairments in signaling, *Arc* gene expression, and synaptic and behavioral outcomes in adult mice. The observation of synaptic deficits is consistent with previous studies reporting that excitatory synaptic transmission is impaired in cultured hippocampal neurons derived from MeCP2 null mice (Nelson et al., 2006; Chao et al., 2007), or vice versa in mice overexpressing MeCP2 (Chao et al., 2007; Na et al., 2012, 2013). Additionally, reduced hippocampal slice LTP and learning and memory behavior is found in MeCP2 null mice (Asaka et al., 2006; Moretti et al., 2006; Guy et al., 2007; Na et al., 2012, 2013). Taken together, our results suggest that MeCP2 plays an essential role in the postnatal ethanol-induced defects in synaptic circuit maturation in the developing brain.

Dynamic modification of DNA methylation and binding partners of methylation machinery, such as MeCP2, occur in a tissue, and time-specific manner and appear to be critically important for early brain development (Matsuda and Yasutomi, 1992; Kakutani et al., 1996; Martin et al., 1999). Ethanol exposure during embryonic development was shown to modulate DNA methylation patterns (Garro et al., 1991; Haycock and Ramsay, 2009; Liu et al., 2009; Ouko et al., 2009; Downing et al., 2011; Zhou et al., 2011), and is implicated in the etiology of numerous developmental abnormalities (Ouko et al., 2009; Kaminen-Ahola et al., 2010). Our recent study (Nagre et al., 2015) suggested that ethanol-induced CB1R-mediated caspase-3 degrades DNMT1 and DNMT3A proteins, causing DNA hypomethylation in P7 mice. Consistent with this observation, in another of our studies (Subbanna et al., 2013b) ethanol-activated caspase-3 in P7 mice cleaves explicitly dimethylated (lysine 9) histone proteins. In another study, direct inhibition of DNA methylation by 5-azacytidine (5-AzaC) at P7 also caused Arc deficits, resulting in neurodegeneration in neonatal mice and long-lasting impaired Arc expression and behavioral defects in adult mice (Subbanna et al., 2016). Together, these observations suggest that postnatal ethanol-activated caspase-3 can impact epigenetic control of gene expression through modulation of MeCP2 and DNMTs for a short period during postnatal development. Thus, this can lead to persistent neurological and behavioral deficits in adult mice (Subbanna et al., 2013a, 2015a; Subbanna and Basavarajappa, 2014), as observed in animal models of FASD (Izumi et al., 2005; Wilson et al., 2011; Sadrian et al., 2012; Subbanna et al., 2013a; Subbanna and Basavarajappa, 2014).

We found a contrasting effect of ethanol treatment of P7 mice on the expression levels of *Mecp2* mRNA and protein. Although a similar discrepancy between MeCP2 protein and *Mecp2* mRNA levels was shown in mouse brain tissues (Shahbazian et al., 2002; Dura et al., 2008), postnatal ethanol exposure may enhance transcription of the MECP2 gene, but robust activation of caspase-3 resulted in proteolytic degradation of MeCP2 protein. In our previous studies (Subbanna et al., 2013b), we showed that dimethylated H3K9 acts as a substrate for activated caspase-3. Similarly, a CB1R antagonist, which is known to inhibit ethanol-induced caspase-3-mediated (Subbanna et al., 2013a) degradation of DNMT1 and DNMT3A proteins and reduce DNA methylation (Nagre et al., 2015) in P7 mice, prevented MeCP2 protein degradation. Furthermore, genetic ablation of CB1R, which provides protection from ethanol-activated caspase-3 (Subbanna et al., 2013a)-mediated degradation of DNMT1 and DNMT3A proteins, and DNA hypomethylation (Nagre et al., 2015) in P7 mice and long-lasting neurobehavioral defects in adulthood (Subbanna et al., 2013a), also revived MeCP2 protein levels that had been lowered by ethanol. These findings may indicate that the observed reduction in MeCP2 protein levels for a short period during a critical window of synaptic maturation (Marchal and Mulle, 2004; Lanore et al., 2010) can have a long-lasting effect on epigenetic regulation and synaptic circuit formation, leading to neurobehavioral defects, as observed in mice (Subbanna et al., 2013a, 2015a; Subbanna and Basavarajappa, 2014) and human FASD (Mattson et al., 2011; Lebel et al., 2012; Norman et al., 2013). This hypothesis is supported by the observation that SR141716A and the use of CB1R KO mice protected against P7 ethanol-mediated caspase-3 activation and persistent behavioral problems in adult mice (Subbanna et al., 2013a,b, 2015a; Subbanna and Basavarajappa, 2014).

The levels of cellular proteins that regulate biological processes are controlled by missionaries that are involved in transcription, translation, post-translational and degradation. Under steady-state conditions, most cellular proteins undergo continuous synthesis, modification and degradation processes to adjust to both internal and environmental stimuli. The presence of many caspase-3 substrates was shown in the nucleus (Fischer et al., 2003), and has been suggested to play a crucial role in the nuclear morphological changes that occur in most cells undergoing apoptosis (Kamada et al., 2005). Although the connection between neurodegeneration and MeCP2 protein stability in the developing brain is lacking, our findings suggest that regulation of MeCP2 turnover might be of importance. Consistent with this suggestion, low-dose ethanol treatment of P7 mice, which induces significantly lower amounts of caspase-3 activation in P7 mice, enhances MeCP2 levels without any proteolytic degradation (Subbanna et al., 2014). Prenatal ethanol exposure during pregnancy also enhanced *Mecp2* gene expression in rats (Bekdash et al., 2013; Perkins et al., 2013). It was shown that chronic ethanol exposure led to lower 5-mc levels and up regulated MeCP2 levels, while ethanol withdrawal reduced expression in embryonic differentiating brain cells (Liyanage et al., 2015). Additionally, prenatal exposure to ethanol during early pregnancy significantly decreased MeCP2 levels in both the prefrontal cortex and striatum of offspring (Kim et al., 2013). Furthermore, fetal alcohol exposure also caused a significant increase in MeCP2 in hypothalamus proopiomelanocortin (POMC) cells as well as at the *Pomc* gene promoter (Gangisetty et al., 2014). Therefore, our current study demonstrates that MeCP2 proteins may serve as substrates for widely activated caspase-3 in ethanol-treated P7 mouse brains. Thus, the caspase-3 inhibitor (Q-VD-OPh) prevents the loss of MeCP2 protein in ethanol-exposed P7 mice. However, our study does not exclude other possible proteolytic mechanisms involved in the loss of MeCP2 in ethanol-treated P7 mice. Similar degradation of MeCP2 was found in ethanol-treated murine embryonic fibroblasts (MEFs; Mukhopadhyay et al., 2013). Additionally, pretreatment of MEFs with the proteosomal inhibitor MG-132 before ethanol exposure completely prevented the degradation of MeCP2 protein (Mukhopadhyay et al., 2013). Previous findings have shown that several RTT mutations are characterized by a decrease in MeCP2 stability (Ballestar et al., 2000; Yusufzai and Wolffe, 2000; Free et al., 2001). MeCP2 was shown to be ubiquitinated by an E3 ubiquitin ligase (RNF4), and this leads to the removal of MeCP2 from gene promoters, leading to activation of transcription (Wang, 2014). The biological function of RNF4 is broad, and it has been shown to target many proteins for degradation (Hu et al., 2010). Future studies of the mechanisms (e.g., ubiquitination and protein degradation) related to MeCP2 protein stability and turnover that lead to an imbalance in MeCP2 protein levels would provide more insight into neurodevelopmental disorders resulting from environmental insults, including FASD. Thus, it is possible that, during early neurodevelopment, ethanol-induced impairments of MeCP2 and DNA methylation (Nagre et al., 2015) may affect the binding of transcription factors to gene promoter regions, leading to suppression of the expression of genes (e.g., *Arc*) that encode survival factor(s) (Kokubo et al., 2009), which may impact neuronal development and neurobehavioral abnormalities, such as deficits in synaptic plasticity, and learning and memory in adult animals (Noel et al., 2011; Wilson et al., 2011; Sadrian et al., 2012; Subbanna et al., 2013a). Indeed, developmental alcohol, including postnatal ethanol exposure, impairs LTP and the performance of several behavioral tasks related to learning and memory (Abel et al., 1983; Bonthius and West, 1991; Bellinger et al., 1999, 2002; Berman and Hannigan, 2000; Alati et al., 2006; Brown et al., 2007; Brady et al., 2013; Subbanna et al., 2013a, 2015a; Subbanna and Basavarajappa, 2014; Basavarajappa, 2015).

Our previous findings indicated the involvement of the ERK1/2-pCREB-Arc pathway, which operates on neuronal survival downstream of the CB1Rs in the developing brain (Subbanna et al., 2013a,b). Ethanol treatment during postnatal development was shown to impair this pathway (Subbanna et al., 2013a, 2015a). The current study replicated these previous findings and suggests that deficits in CREB activation and Arc expression last into adulthood and can be rescued by either pre-administration of CB1R antagonist or a caspase-3 inhibitor before P7 ethanol treatment. In addition, by using a ChIP assay, we demonstrated a marked increase in H3K9me2 and a reduction in H3K14ace at the *Arc* gene promoter. Loss of MeCP2 could silence Arc gene transcription by recruiting HDAC (Nan et al., 1997, 1998) or G9a (Fuks et al., 2003; Coward et al., 2017), both of which have been shown to remove acetyl groups from histones or methylate histones respectively, resulting in gene suppression. Additionally, reduced pCREB levels can impair CBP (HAT) recruitment to gene promoters (Lonze and Ginty, 2002; Lubin et al., 2008), leading to reduced acetylation of histone proteins. Together, these events can alter H3K9me2 and H3K14ace (Basavarajappa and Subbanna, 2016) to repress Arc expression. These findings provide epigenetic mechanisms for the P7 ethanol-induced long-lasting Arc expression deficits, which are a significant part of synaptic function and cognitive behavior (Donai et al., 2003; Chowdhury et al., 2006; Bramham et al., 2008; Kawashima et al., 2009; Korb and Finkbeiner, 2011; Yamada et al., 2011; Dyrvig et al., 2012). It should be emphasized here that ethanol-induced inhibition of the CB1R-regulated ERK1/2-pCREB-Arc signaling pathway might disrupt the proper development of synaptic circuits, leading to lasting synaptic, learning and memory defects (Abel et al., 1983; Bonthius and West, 1991; Bellinger et al., 1999, 2002; Berman and Hannigan, 2000; Alati et al., 2006; Brown et al., 2007; Brady et al., 2013; Subbanna et al., 2013a, 2015a; Subbanna and Basavarajappa, 2014; Basavarajappa, 2015). Similarly, CB1RKO mice do not portray P7 ethanol-induced neurodegeneration or a loss of DNMTs (Hansen et al., 2008; Subbanna et al., 2013a; Basavarajappa, 2015; Nagre et al., 2015) and MeCP2 during the neonatal period or deficits in LTP, learning and memory during adulthood (Subbanna et al., 2013a; Basavarajappa, 2015; Nagre et al., 2015). These observations together suggest that signaling events and epigenetic changes that occur during the neurodegeneration process in the developing brain are crucial for understanding the etiology of FASD.

Our data suggests that P7 ethanol-treated adult mice exposed to Y-maze behavior exhibit deficits in activity-dependent signaling events, such as activation of CaMKIV and CREB, and formation of Arc. We also found reduced pCaMKIV, pCREB, pMeCP2 and Arc expression in P7 ethanol-treated adult mice not exposed to Y-maze behavior (basal deficits). Although Y-maze behavior failed to enhance pMeCP2 in P7 saline-treated adult mice, it was successful in improving pMeCP2 in P7 ethanol-treated adult mice. A previous study using cultured cortical neurons suggested that loss of pMeCP2 leads to impaired dendritic and synaptic development (Cohen et al., 2011). Using slices containing the visual cortex from P16 to P17 mice, it was shown that activity-dependent activation of MeCP2 through phosphorylation is necessary for the proper maturation of cortical synaptic circuits (Cohen et al., 2011). It should be noted that activity-dependent changes in the neurons contribute to the adequate maturation of neural circuits during development (Hong et al., 2005). Furthermore, activity-dependent changes in signaling events and gene transcription are required in the persistent refinement of neural circuits (Arancio et al., 1995; Taha and Stryker, 2002; Lu, 2003; Tabuchi, 2008; Kawashima et al., 2009). Interestingly, neural activity during development has been shown to regulate the activity of many genes (West and Greenberg, 2011), including Arc gene expression, through several signaling events (Plath et al., 2006; Rial Verde et al., 2006; Chotiner et al., 2010). Whether or not such regulation is under the control of MeCP2 phosphorylation needs to be investigated in future studies. Nevertheless, the observed activity-dependent impairments that are important for stabilizing the activity-dependent refinement of the neural circuits (Wong and Ghosh, 2002; Van Aelst and Cline, 2004; Fox and Wong, 2005) could also lead to persistent learning and memory deficits, as observed in animal models of FASD (Abel et al., 1983; Bonthius and West, 1991; Bellinger et al., 1999, 2002; Berman and Hannigan, 2000; Alati et al., 2006; Brown et al., 2007; Brady et al., 2013; Subbanna et al., 2013a, 2015a; Subbanna and Basavarajappa, 2014; Basavarajappa, 2015). The previous (Subbanna et al., 2013a, 2015a) and current findings together strongly suggest that characterization of CB1R downstream targets that regulate pCREB-mediated epigenetic events, gene expression, and the effects of developmental (postnatal) ethanol exposure may ultimately aid in the development of therapeutic drugs to reduce the symptoms of developmental ethanol neurobehavioral teratogenicity.

**Figure 7:**
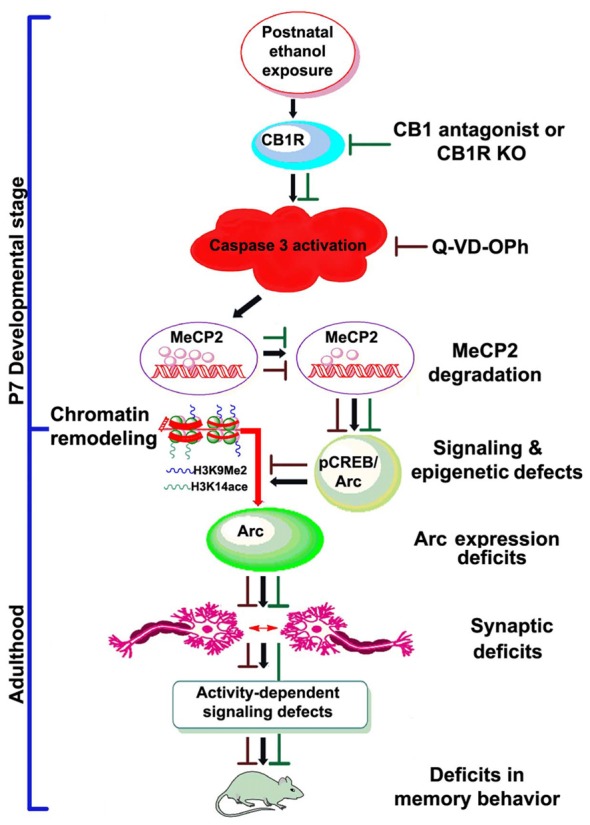
Schematic diagram showing the molecular mechanism by which CB1R-mediated activation of caspase-3 degrades MeCP2, leading to neurobehavioral abnormalities in postnatal ethanol-exposed mice. It was revealed previously that P7 ethanol treatment activates CB1R and inhibits pCREB and Arc expression, leading to neurodegeneration in neonatal mice and persistent neurobehavioral abnormalities in adult mice (Subbanna et al., 2015a). Here, we show that inhibition of CB1R (Subbanna et al., 2013a, 2014) or caspase-3 prevents loss of MeCP2 and rescues pCREB/Arc defects in neonatal mice. Inhibition of caspase-3 in P7 mice also protected against the synaptic plasticity, Arc expression, activity-dependent signaling and behavioral defects in adult mice exposed to ethanol at P7. These observations suggest that CB1R (Subbanna et al., 2013a, 2014)/caspase-3-mediated MeCP2 loss in early development causes neurobehavioral abnormalities in postnatal ethanol-exposed adult mice (↓, ethanol effects; ⊥, drug effects).

In conclusion, the present study presents several lines of evidence indicating that postnatal ethanol treatment reduces the levels of MeCP2 protein in a CB1R-mediated caspase-3-dependent manner, which has a crucial role in gene expression regulation. The experimental evidence in the current study provides novel mechanistic insights into the developmental effects of ethanol and indicate that ethanol may disrupt MeCP2 proteins, leading to persistent deficits in *Arc* gene expression and synaptic impairments (Figure 7). These events occur within the developing brain and are responsible for persistent neurobehavioral impairments similar to those observed in FASD.

## Author Contributions

BSB conceived, designed the experiments and contributed to study supervision. BSB, SS, NNN, MS, NSU, AK, VJ and DP performed the experiments and contributed to data analysis. BSB and SS wrote the manuscript.

## Conflict of Interest Statement

The authors declare that the research was conducted in the absence of any commercial or financial relationships that could be construed as a potential conflict of interest.
